# Supplementary material for: A Humanized Yeast Phenomic Model of Deoxycytidine Kinase to Predict Genetic Buffering of Nucleoside Analog Cytotoxicity
Source: Genes (Basel). 2019 Sep 30;10(10):770. doi: 10.3390/genes10100770 (PMC6826991; doi:10.3390/genes10100770)
Supplement: Supplementary file 1 [file genes-10-00770-s001.zip › Additional_Files/Additional_File7_GOTermHeatmaps.zip!/Additional_File7_GOTermHeatmaps/RNA_polymerase_II_transcription_factor_complex.pdf]

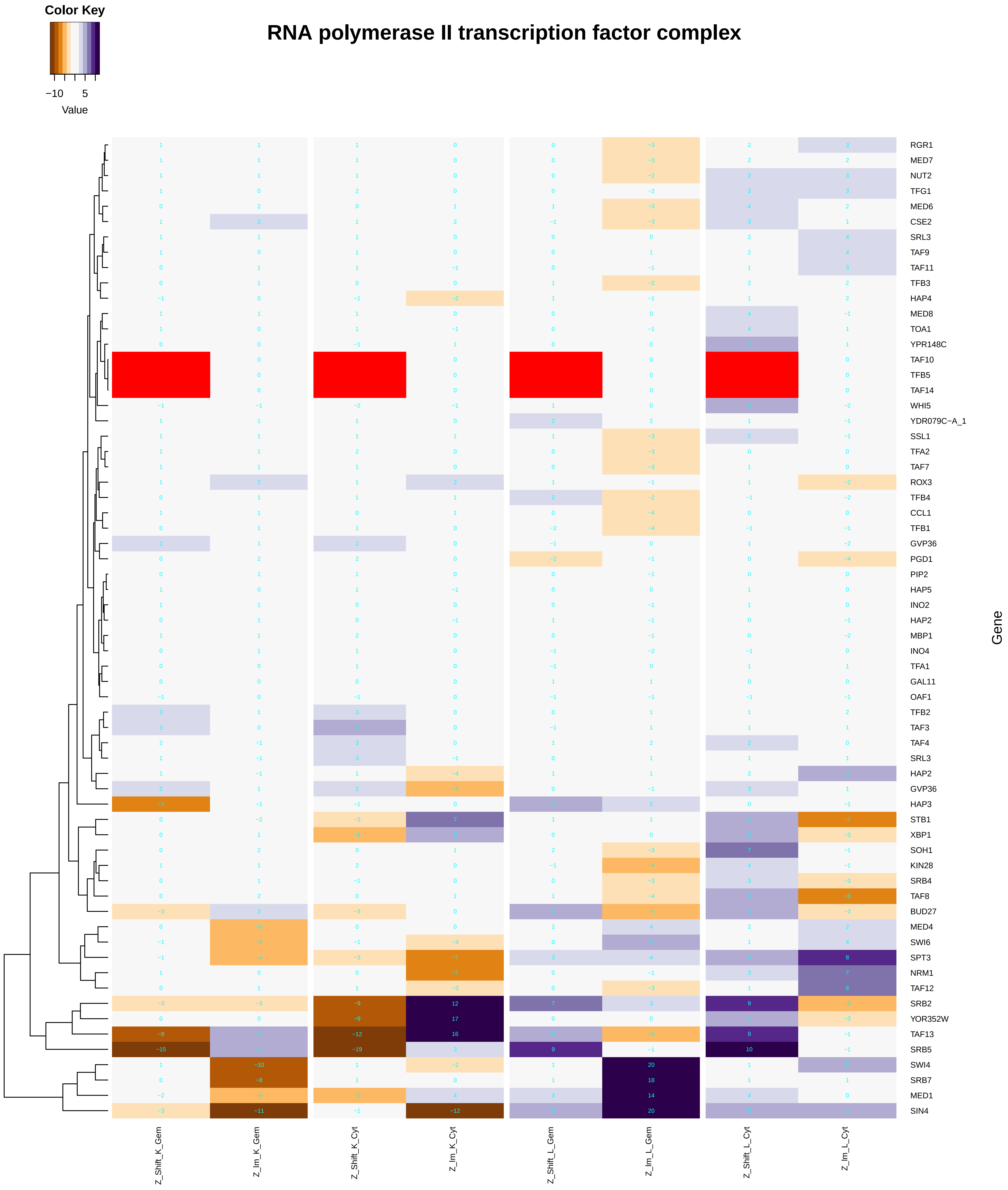
RNA polymerase II transcription factor complex
Color Key
−10
5
Value
RGR1
MED7
NUT2
TFG1
MED6
CSE2
SRL3
TAF9
TAF11
TFB3
HAP4
MED8
TOA1
YPR148C
TAF10
TFB5
TAF14
WHI5
YDR079C−A_1
SSL1
TFA2
TAF7
ROX3
TFB4
CCL1
TFB1
GVP36
PGD1
PIP2
HAP5
INO2
HAP2
MBP1
INO4
TFA1
GAL11
OAF1
TFB2
TAF3
TAF4
SRL3
HAP2
GVP36
HAP3
STB1
XBP1
SOH1
KIN28
SRB4
TAF8
BUD27
MED4
SWI6
SPT3
NRM1
TAF12
SRB2
YOR352W
TAF13
SRB5
SWI4
SRB7
MED1
SIN4
Gene
Z_Shift_K_Gem
Z_Im_K_Gem
Z_Shift_K_Cyt
Z_Im_K_Cyt
Z_Shift_L_Gem
Z_Im_L_Gem
Z_Shift_L_Cyt
Z_Im_L_Cyt

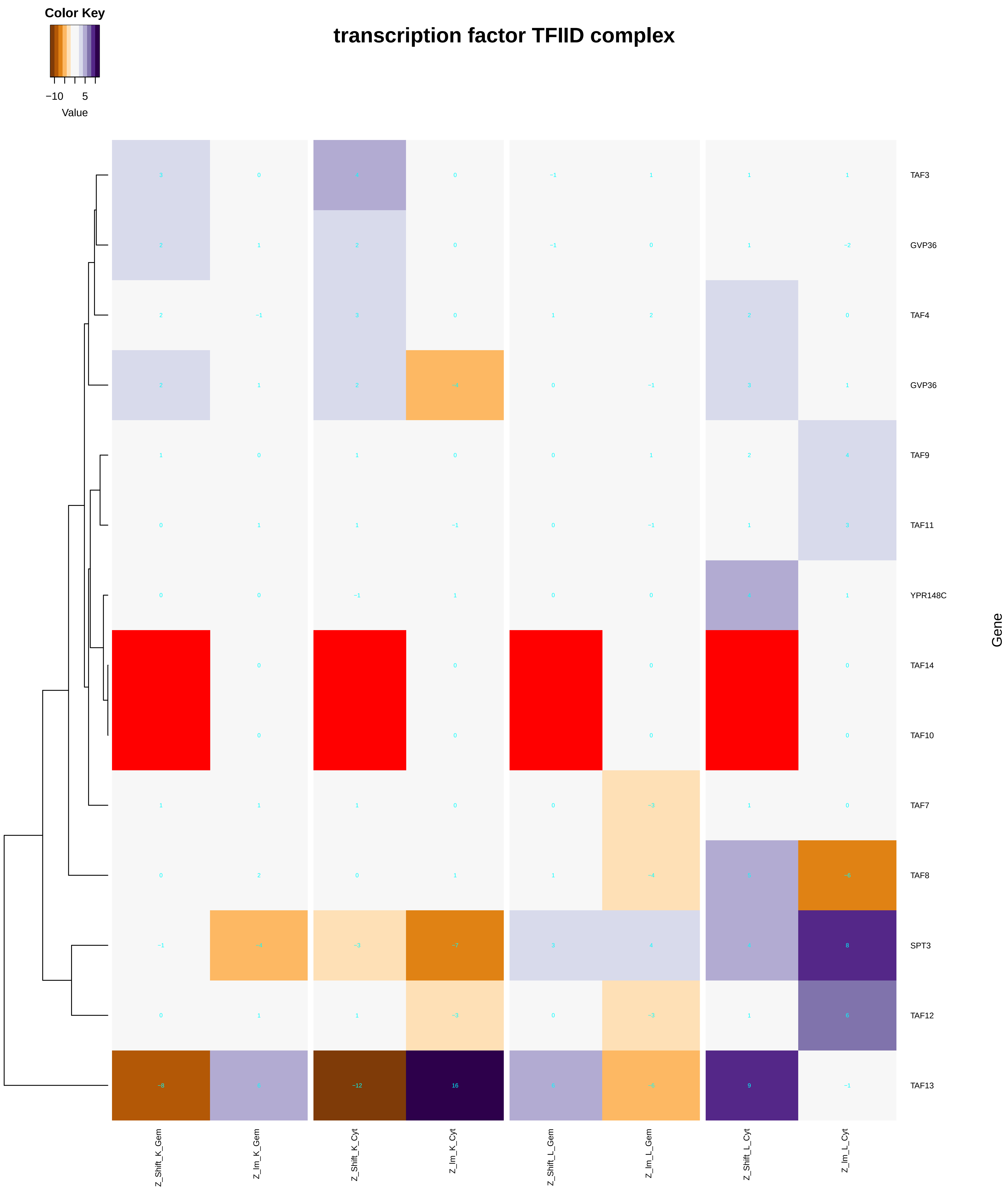

Color Key
−10
5
Value
transcription factor TFIID complex
TAF3
GVP36
TAF4
GVP36
TAF9
TAF11
YPR148C
TAF14
TAF10
TAF7
TAF8
SPT3
TAF12
TAF13
Gene
Z_Shift_K_Gem
Z_Im_K_Gem
Z_Shift_K_Cyt
Z_Im_K_Cyt
Z_Shift_L_Gem
Z_Im_L_Gem
Z_Shift_L_Cyt
Z_Im_L_Cyt

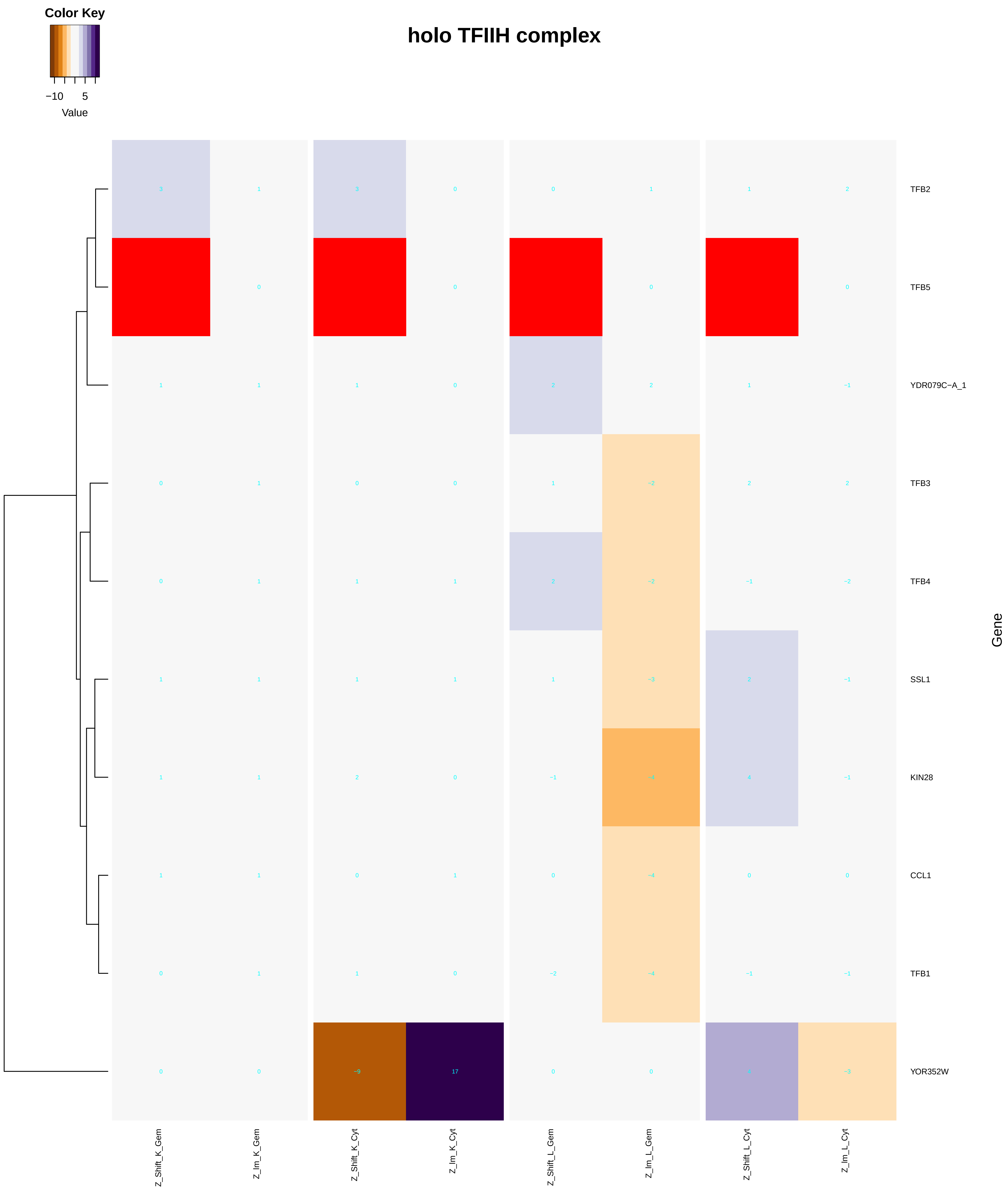
Color Key
−10
5
Value
holo TFIIH complex
TFB2
TFB5
YDR079C−A_1
TFB3
TFB4
SSL1
KIN28
CCL1
TFB1
YOR352W
Gene
Z_Shift_K_Gem
Z_Im_K_Gem
Z_Shift_K_Cyt
Z_Im_K_Cyt
Z_Shift_L_Gem
Z_Im_L_Gem
Z_Shift_L_Cyt
Z_Im_L_Cyt

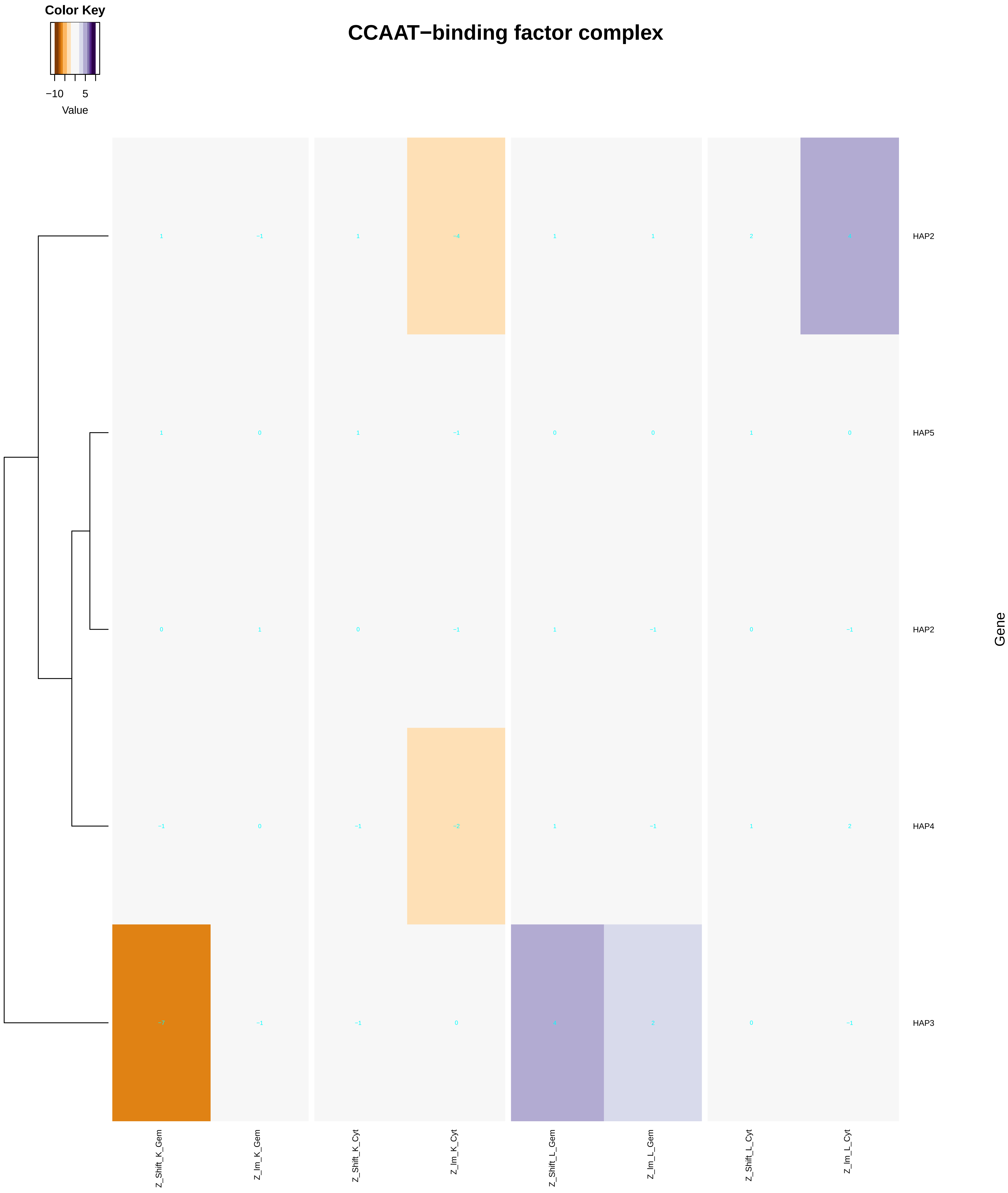
Color Key
−10
5
Value
CCAAT−binding factor complex
HAP2
HAP5
HAP2
HAP4
HAP3
Gene
Z_Shift_K_Gem
Z_Im_K_Gem
Z_Shift_K_Cyt
Z_Im_K_Cyt
Z_Shift_L_Gem
Z_Im_L_Gem
Z_Shift_L_Cyt
Z_Im_L_Cyt

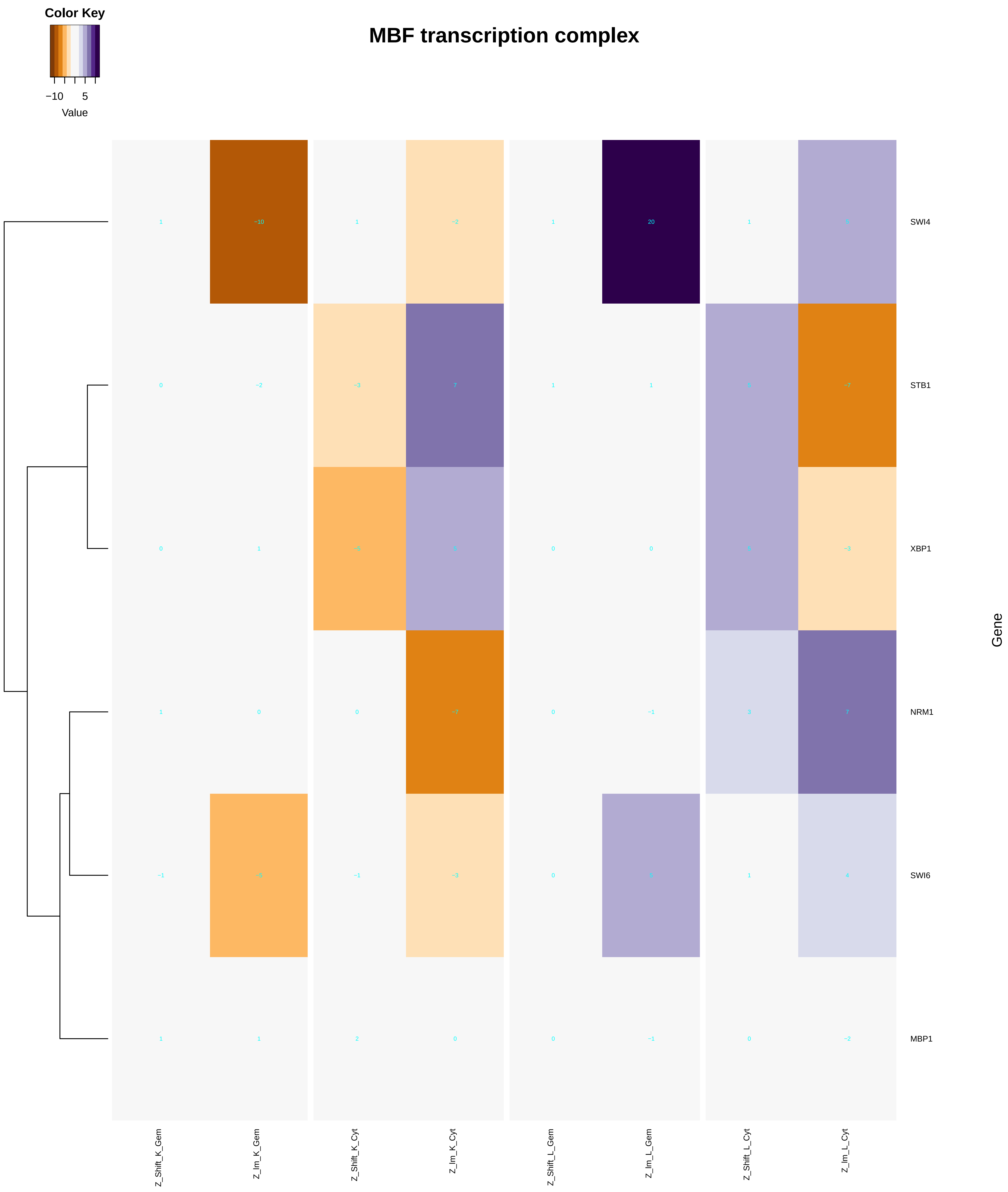
Color Key
−10
5
Value
MBF transcription complex
SWI4
STB1
XBP1
NRM1
SWI6
MBP1
Gene
Z_Shift_K_Gem
Z_Im_K_Gem
Z_Shift_K_Cyt
Z_Im_K_Cyt
Z_Shift_L_Gem
Z_Im_L_Gem
Z_Shift_L_Cyt
Z_Im_L_Cyt

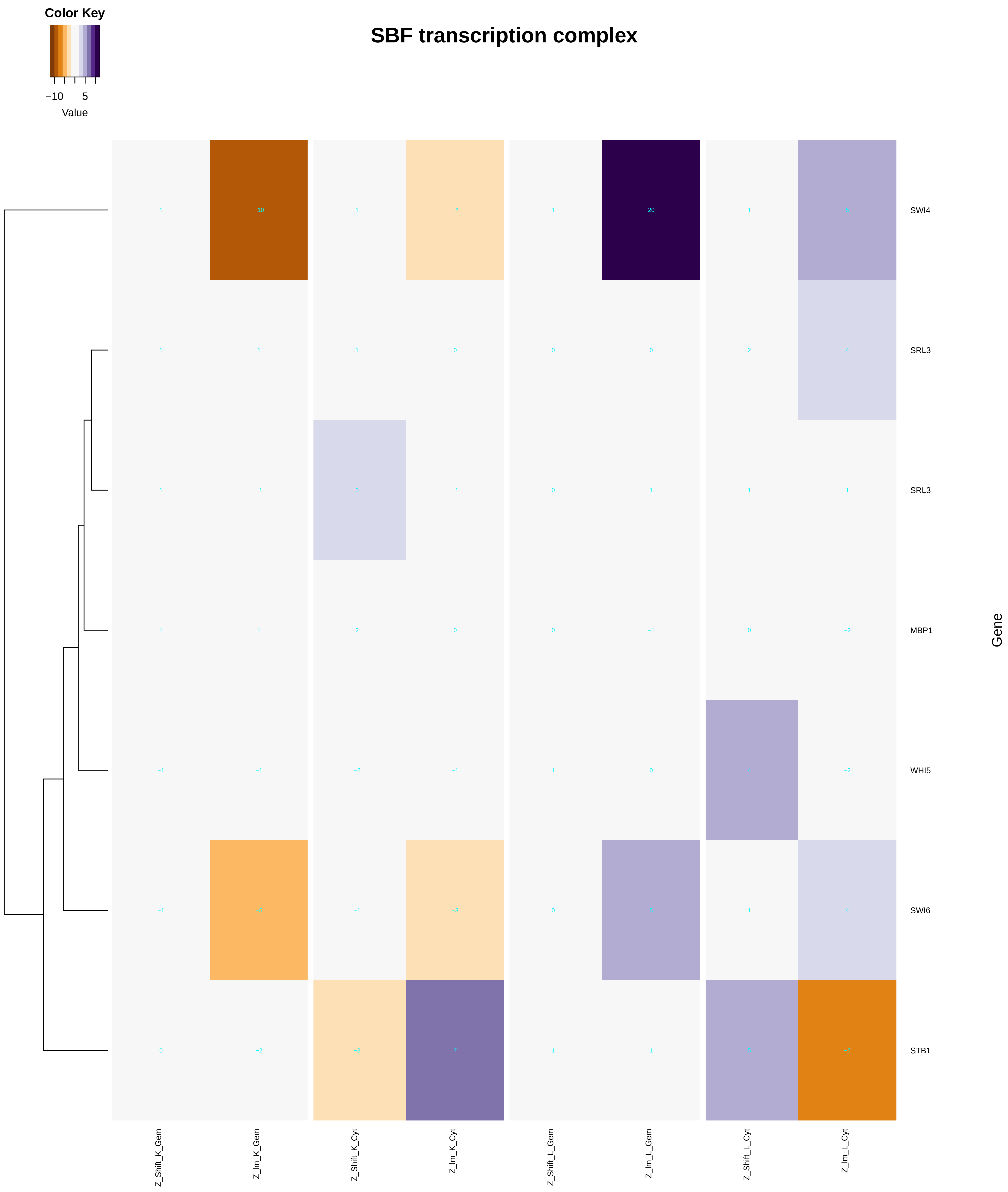
Color Key
−10
5
Value
SBF transcription complex
SWI4
SRL3
SRL3
MBP1
WHI5
SWI6
STB1
Gene
Z_Shift_K_Gem
Z_Im_K_Gem
Z_Shift_K_Cyt
Z_Im_K_Cyt
Z_Shift_L_Gem
Z_Im_L_Gem
Z_Shift_L_Cyt
Z_Im_L_Cyt

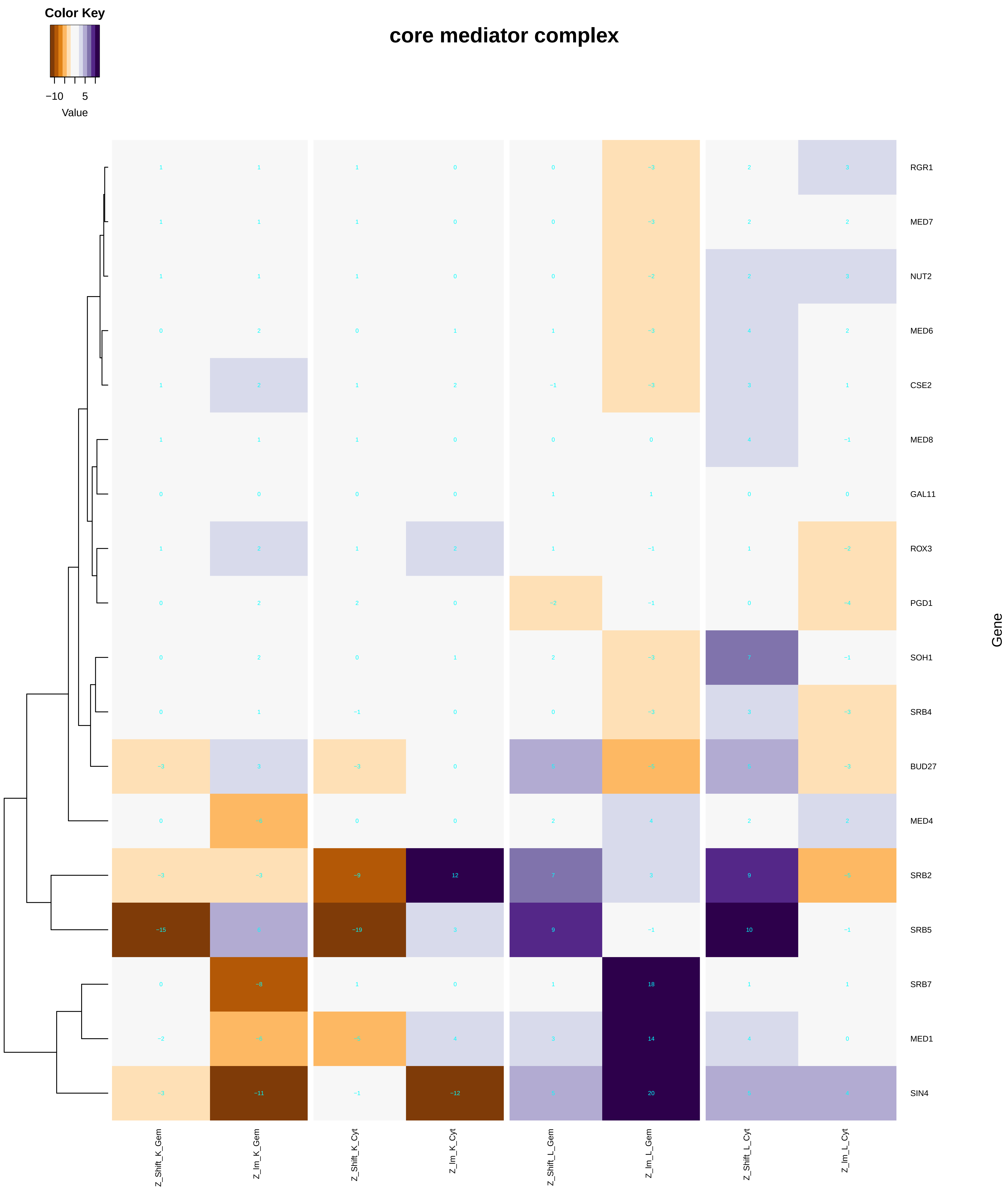

Color Key
−10
5
Value
core mediator complex
RGR1
MED7
NUT2
MED6
CSE2
MED8
GAL11
ROX3
PGD1
SOH1
SRB4
BUD27
MED4
SRB2
SRB5
SRB7
MED1
SIN4
Gene
Z_Shift_K_Gem
Z_Im_K_Gem
Z_Shift_K_Cyt
Z_Im_K_Cyt
Z_Shift_L_Gem
Z_Im_L_Gem
Z_Shift_L_Cyt
Z_Im_L_Cyt

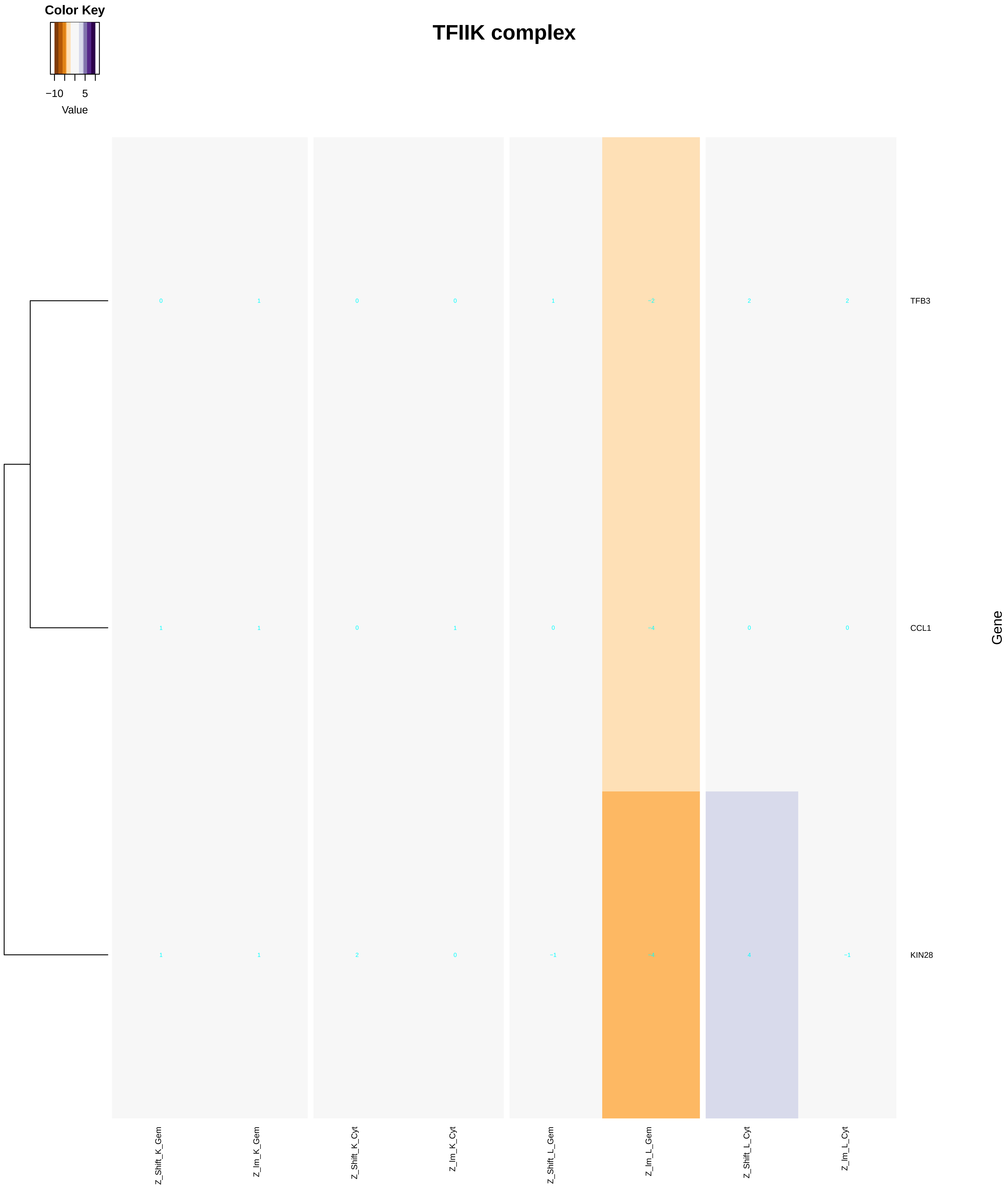
Color Key
−10
5
Value
TFIIK complex
0
1
0
0
1
−2
2
2
TFB3
1
1
0
1
0
−4
0
0
CCL1
1
1
2
0
−1
−4
4
−1
KIN28
Gene
Z_Shift_K_Gem
Z_Im_K_Gem
Z_Shift_K_Cyt
Z_Im_K_Cyt
Z_Shift_L_Gem
Z_Im_L_Gem
Z_Shift_L_Cyt
Z_Im_L_Cyt
